# Emerging Immune Checkpoint Molecules on Cancer Cells: CD24 and CD200

**DOI:** 10.3390/ijms242015072

**Published:** 2023-10-11

**Authors:** Sun Young Moon, Minjoo Han, Gyoungah Ryu, Seong-Ah Shin, Jun Hyuck Lee, Chang Sup Lee

**Affiliations:** 1 College of Pharmacy and Research Institute of Pharmaceutical Sciences, Gyeongsang National University, Jinju 52828, Republic of Korea; symoon0414@gnu.ac.kr (S.Y.M.); dlsel79@gnu.ac.kr (M.H.); rga_97@gnu.ac.kr (G.R.); shinsaya@gnu.ac.kr (S.-A.S.); 2 Research Unit of Cryogenic Novel Material, Korea Polar Research Institute, Incheon 21990, Republic of Korea; junhyucklee@kopri.re.kr; 3 Department of Polar Sciences, University of Science and Technology, Incheon 21990, Republic of Korea

**Keywords:** immune checkpoint molecules, CD24, CD200, Siglec-10, CD200 receptor

## Abstract

Cancer immunotherapy strategies are based on the utilization of immune checkpoint inhibitors to instigate an antitumor immune response. The efficacy of immune checkpoint blockade, directed at adaptive immune checkpoints, has been demonstrated in select cancer types. However, only a limited subset of patients has exhibited definitive outcomes characterized by a sustained response after discontinuation of therapy. Recent investigations have highlighted the significance of immune checkpoint molecules that are overexpressed in cancer cells and inhibit myeloid lineage immune cells within a tumor microenvironment. These checkpoints are identified as potential targets for anticancer immune responses. Notably, the immune checkpoint molecules CD24 and CD200 have garnered attention owing to their involvement in tumor immune evasion. CD24 and CD200 are overexpressed across diverse cancer types and serve as signaling checkpoints by engaging their respective receptors, Siglec-10 and CD200 receptor, which are expressed on tumor-associated myeloid cells. In this review, we summarized and discussed the latest advancements and insights into CD24 and CD200 as emergent immune checkpoint moieties, further delving into their therapeutic potentials for cancer treatment.

## 1. Introduction

Cancer is a perpetually advancing disease characterized by the development of abnormal cells that are uncontrollably divided [1]. Despite advancements in anticancer therapies, cancer remains one of the leading causes of mortality [2]. The immune system substantially affects the development of cancer cells and the pertinent treatment approaches. Cancer immunotherapy involves multiple immunomodulatory strategies to control the progression of malignant tumors. Recently, immunotherapy via immune checkpoint blockade has been successfully used to treat several cancer types [3]. 

Immune checkpoints include costimulatory molecules, such as CD28, and co-inhibitory signaling molecules, including cytotoxic T-lymphocyte-associated protein 4 (CTLA-4) and programmed cell death protein 1 (PD-1), which are required for immune homeostasis [4]. These checkpoints influence the balance between costimulation and co-inhibition, facilitating self-tolerance under physiological conditions. However, tumor cells exploit immune checkpoint pathways to evade immune surveillance, thereby suppressing antitumor immune responses [5]. Immune checkpoint molecules, such as CTLA-4 and PD-1, disrupt antitumor immunity by attenuating T-cell activation in the event of malignancy, leading to a highly immunosuppressive tumor microenvironment [6]. Immune checkpoint inhibitors (ICIs) targeting PD-1, such as nivolumab, cemiplimab, and pembrolizumab, and those targeting CTLA-4, such as ipilimumab, have been clinically approved for the treatment of several cancer types [7]. While these immune checkpoint blockades have been shown to augment host immune responses against cancer by targeting T-cell immune checkpoints, they also directly or indirectly regulate innate immune cells [8]. The therapeutic efficacy of the currently used immune checkpoints is restricted to relatively few patients with certain types of cancer, and the majority still do not receive benefits [9]. Consequently, there has been increasing interest in new ICIs that directly target innate immune checkpoints extending beyond those targeting adaptive immune checkpoints that generate T-cell activation against cancer [10].

**Figure 1:**
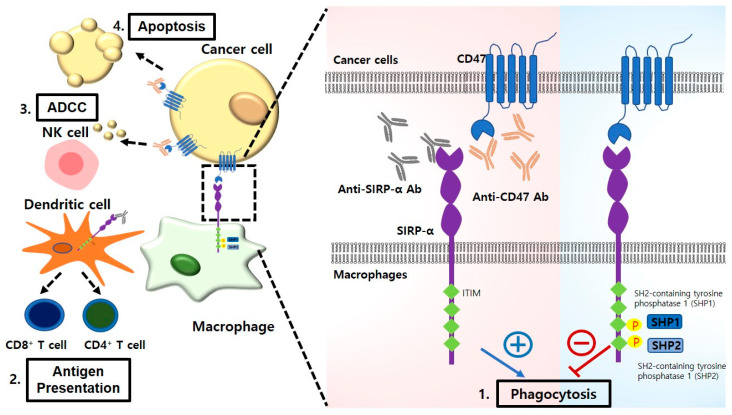
Interaction of CD47 on cancer cells with SIRPα on phagocytes. Innate immune checkpoint CD47–SIRPα axis can be targeted through multiple mechanisms (1–4) in the tumor microenvironment. 1. CD47–SIRPα binding induces the phosphorylation of two immunoreceptor tyrosine-based inhibition motifs (ITIMs) in the cytoplasmic tail of SIRPα. This leads to the recruitment and activation of phosphatases, including SHP1 and SHP2, ultimately resulting in the inhibition of cancer cell phagocytosis by macrophages. Anti-CD47 antibody or anti-SIRPα antibody induces the uptake of tumor cells by macrophages via blocking the interaction between “Don’t eat me” signal (CD47) and immune checkpoint receptor (SIRPα). 2. Anti-CD47 antibodies facilitate phagocytic absorption of cancer cells by dendritic cells. This triggers an anticancer adaptive immune response. 3. Anti-CD47 antibodies eradicate cancer cells via natural killer antibody-dependent, cell-mediated cytotoxicity. ADCC: antibody-dependent, cell-mediated cytotoxicity. 4. Anti-CD47 antibodies induce apoptosis in cancer cells.

In cancer, antigen-presenting cells, such as macrophages and dendritic cells, phagocytose cancer cells and present cancer-specific antigens to T cells to prime them, thereby generating cancer-specific T cells [11]. However, cancer cells avoid phagocytosis by upregulating “Don’t eat me” signaling molecules, and the interaction between the “Don’t eat me” ligand and its counter receptor helps tumor cells to escape phagocytic uptake [12]. CD47, identified as a self-marker on RBCs to prevent their clearance by macrophages, is overexpressed in most types of cancer cells and is considered a tumor phagocytosis checkpoint molecule [13]. CD47 on cancer cells interacts with the inhibitory receptor signal regulatory protein alpha (SIRPα), which is expressed in phagocytes. Targeting CD47–SIRPα can eliminate cancer cells through multiple mechanisms (Figure 1) [14,15]. Ongoing clinical studies have been conducted to inhibit the CD47–SIRPα axis using several antibodies or fusion proteins targeting CD47 and SIRPα in some solid and hematologic cancers [16]. Increasing evidence has suggested that blocking CD47–SIRPα interaction promotes the phagocytosis of cancer cells, resulting in the suppression of tumor growth and progression. 

To date, many immune checkpoint inhibitors (ICIs) have been successfully used to treat various types of cancer. Although ICIs are clinically effective for particular cancer types, the rapid development of resistance occurs in many patients [17]. Furthermore, limited response to immunotherapy has been observed in various studies, resulting from the complex redundant mechanisms of cancer-mediated immune repression [18]. Given the dynamic complexity of the host–immune tumor interaction, there is a need for research on a novel immune checkpoint signaling axis between tumor and immune cells for more effective anticancer immunotherapy. Drawing from the multiple scientific studies on cancer immunotherapy, we narrowed our focus to CD24 and CD200 as immune checkpoint molecules that are overexpressed in cancer cells. After describing the association between CD24 and CD200 expression and tumor progression, we conducted a detailed exploration into the immunosuppressive effects of these molecules. Our emphasis was on illustrating how CD24 and CD200 act as signaling checkpoints by engaging their respective receptors, namely, Siglec-10 and CD200 receptor, which are expressed on tumor-associated myeloid cells. In the present review, we summarize the recent progress and understanding of CD24 and CD200 as emerging immune checkpoint signals in cancer as well as their interaction with their cognate receptors, sialic acid-binding immunoglobulin (Ig)-like lectin 10 (Siglec-10) and CD200 receptor (CD200R). Furthermore, we discuss their potential as immunotherapeutic targets for cancer treatment.

## 2. CD24

CD24, first identified as a heat-stable antigen (HAS) because of its ability to resist heat, is a sialic acid glycoprotein with multiple O- and N-glycosylation sites [19]. CD24 is a cell-surface protein anchored to the plasma membrane via glycosyl-phosphatidyl-inositol (GPI) [20]. CD24 is predominantly expressed on the surface of immune cells such as T and B lymphocytes and granulocytes. It is also expressed in epithelial cells, neural cells, muscle cells, and many types of cancer cells [19,21]. CD24 is highly expressed in stem-like progenitor cells or metabolically active proliferative cells and may play a role in the differentiation of various cell types [22,23,24]. CD24 overexpression in multiple cancer cell types is associated with the development and progression of cancer, resulting in poor prognosis [25,26]. Notably, the high expression of CD24 on the cancer cell surface acts as an innate immune checkpoint molecule that inhibits phagocytosis during the interaction between immune cells and cancer cells, leading to tumor-mediated immune escape [27].

### 2.1. CD24 Expression in Tumor Cells and Its Effects on Tumor Progression

CD24 expression is evident across various human tumor cells, and extensive studies have highlighted the association between its overexpression with tumor formation and progression [28]. Notably, CD24 expression correlated with poor prognosis in several cancer types, including solid tumors and hematologic malignancies [29].

CD24 expression was detected in human breast cancer cell lines, and the potency of its expression correlated with breast tumor grade [30]. CD24 expression is also closely associated with poor prognosis in patients with breast cancer [31,32]. Notably, human epidermal growth factor receptor 2 (HER2)-positive breast cancer cells highly express CD24, and CD24 knockdown increases the susceptibility of these cells to lapatinib, a HER2 inhibitor [33]. Some clinical studies have reported that CD24 expression is closely related to a worsening prognosis in hormone receptor-positive breast cancer and that it could be involved in the less advantageous response of ductal breast cancer to tamoxifen, a competitive estrogen inhibitor [34,35]. CD24 is highly expressed in invasive ovarian cancers but not in normal tissue or benign ovarian tumors, which is related to the reduced survival rate of patients with ovarian cancer [36]. Nakamura et al. discovered that CD24 plays a role in metastatic progression by inducing epithelial–mesenchymal transition in ovarian cancer. It is associated with cisplatin resistance, thereby underscoring its potential as a therapeutic target for advanced ovarian cancer [37]. In endometrial cancers, CD24-positive cells exhibited increased resistance to chemotherapy [38]. CD24 overexpression is associated with advanced metastasis in other malignant cancer types, including cervical [39], esophageal [40], gastric [41,42,43], colon [44], lung [45], hepatic [46], and pancreatic cancers [47,48]. Specifically, in colorectal cancers, CD24 upregulation reportedly occurs at an early stage during colorectal cancer progression [49,50].

CD24 has been described as a biomarker of B-cell development. Its expression levels are low in the earliest stage, increase until the pre-B cell stage, and decrease in mature B cells [22,51]. Typically, elevated CD24 expression is associated with cancer progression and poor prognosis in hematologic cancers such as B cell-derived lymphoma and multiple myeloma (MM) [52,53,54].

### 2.2. CD24 as “Don’t Eat Me” Signal and Interaction with Siglec-10 

Cancer cells employ various immunosuppressive mechanisms to evade immune surveillance [55], thereby facilitating tumor progression and expansion, providing resistance to immune detection and elimination. CD24 has been highlighted as a novel “Don’t eat me” signal that serves as an innate immune checkpoint. CD24 on the surface of cancer cells binds to Siglec-10 on tumor-associated macrophages (TAMs) to prevent phagocytosis [56]. This binding event initiates an inhibitory signaling pathway within TAMs through the CD24–Siglec-10 axis, thereby aiding in immune evasion and promoting tumor growth. 

**Figure 2:**
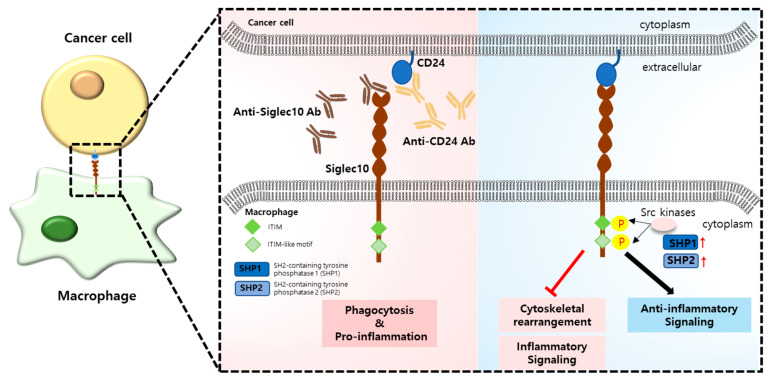
Interaction between CD24 on cancer cells and Siglec-10 on immune macrophages. The binding of CD24 to Siglec-10 induces Src kinases through ITIM and ITIM-like motifs, leading to the phosphorylation of ITIM tyrosine and the recruitment of tyrosine phosphatases (SHP1 and SHP2). These inhibitory signaling cascades block cytoskeletal rearrangement and inflammatory signaling. Blocking the CD24–Siglec-10 axis using anti-CD24 or anti-Siglec-10 antibodies induces phagocytosis and proinflammation in macrophages.

Siglecs are type I transmembrane receptors belonging to the immunoglobulin superfamily. Many siglecs are inhibitory receptors containing immune receptor tyrosine inhibitory motifs (ITIMs) or ITIM-like motifs in their cytoplasmic tail [57]. Siglec activation by specific ligands induces the phosphorylation of ITIM or ITIM-like motif tyrosines by Src family kinases and recruits protein tyrosine phosphatases such as the Src homology-2 domain (SH2)-containing SHP-1 and SHP-2. These phosphatases play downstream effector roles in transducing inhibitory signals (Figure 2) [58,59]. Siglecs interact with sialic acid-containing ligands, such as sialylated pathogens, which contributes to the inhibition of host innate immune cells for immune evasion [60]. Siglec-10 is a member of the Siglec family, which is a group of structurally related cell-surface glycan-binding proteins [61,62]. CD24, a severely sialylated protein, is highly expressed in various cancer cells and is recognized by Siglec-10 on immune cells such as macrophages [63]. Siglec-10 acts as an antiphagocytic receptor by binding to CD24. CD24 modulates the immune response to multiple tumor types by interacting with Siglec-10 [27,56]. CD24 overexpression in ovarian and breast cancer cells acts as an antiphagocytic signal by interacting with Siglec-10. Treatment with an anti-CD24 antibody was found to promote the phagocytic clearance of cancer cells by macrophages by blocking CD24–Siglec-10 interaction [56]. CD24 antibody treatment improved the phagocytosis of patient-derived mantle cell lymphoma (MCL) cells by macrophages but was less effective in diffuse large B-cell lymphoma (DLBCL) [64]. Another study demonstrated that elevated CD24 expression in oral squamous cell carcinoma (OSCC) was correlated with the quantity of TAMs [65]. CD24 blockade in squamous cell-bearing mice has also been confirmed to reduce TAM and tumor growth in hematological malignancy [66]. In addition to CD24 blockade, the recombinant human Siglec-10 Fc chimera decreased anti-inflammatory molecules and considerably increased cytotoxic CD8^+^ cells by blocking Siglec-10 in hepatocellular carcinoma (HCC)-derived single cells. Furthermore, treatment with pembrolizumab, an antibody targeting PD-1, synergistically promoted apoptosis of tumor cells in HCC samples [67].

### 2.3. Immune-Therapeutics Targeting CD24 (CD24-Based Immunotherapy)

To date, many studies have shown that CD24 is abundantly expressed in various human cancers and is correlated with a poor prognosis [28]. Therefore, CD24 inhibition is a promising strategy for cancer therapy. Various anti-CD24-based cancer therapies have been evaluated in preclinical models (Table 1). 

Currently, monoclonal antibody-mediated therapy is widely used to directly target unique or overexpressed antigens on various cancer cells [68]. Several monoclonal antibodies targeting CD24 have been preclinically investigated in various tumor models. CD24-antagonizing antibody SWA11 inhibits tumor growth in vivo in multiple human cancer cell lines, including lung (A549), ovarian (SKOV3ip), pancreatic (BxPC3), and colorectal (HT29) cancer cell lines, in xenograft mouse models [69,70,71]. Furthermore, this antibody blocked MM disease progression by inhibiting cell growth [72]. Anti-CD24 mAb ALB9 reduced lung metastasis in the highly metastatic bladder and breast cancers and prolonged survival [73,74]. Another anti-CD24 antibody, clone SN3, inhibited tumor growth by promoting the macrophage-based phagocytosis of ovarian and breast cancer cells and increased the phagocytosis of mantle cell lymphoma cell lines by M2-like macrophages [56]. G7mAb also inhibited tumor growth by enhancing the anticancer effect of cetuximab in nude mouse xenograft models of lung, liver, and colorectal cancers [75].

Various methods, including recombinant bispecific antibodies, chimeric antigen receptor T cells (CAR-T cells), and antibody–drug conjugates, have been developed to target CD24. The bispecific antibody cG7-MICA targets both the natural killer (NK) cell receptor NK group 2, member D (NKG2D) ligand MHC class I-related chain A (MICA), and CD24, which reduces tumor volume and improves survival rates in Huh-7-bearing nude mice [76]. 

CAR-T cell therapy has been actively investigated as cancer immunotherapy with immune checkpoint blockade [77]. Engineered T cells specific for CD24 slow tumor growth and prolong survival in SCID mice xenografted with human pancreatic carcinoma [78]. NK cells were transduced with an anti-CD24 CAR containing a highly active single-chain variable fragment (scFv) against CD24, which specifically killed patient-derived ovarian cancer cells [79]. Preclinical studies have also demonstrated promising results using antibody–drug conjugates. SWA11.dgA, a conjugate of the anti-CD24 monoclonal antibody SWA11 and deglycosylated ricin A-chain (dgA), improved the survival of SCID mice bearing BL-38 Burkitt’s lymphoma cells [80]. The immunotoxin SWA11-ZZ-PE38, which contains a *Pseudomonas* exotoxin derivative (PE38), reduced HT-29 xenograft tumor volume in mice [81]. Conjugates of anti-CD24 antibody (G7mAb), nitric oxide (NO), or doxorubicin (DOX) inhibit the growth of hepatocellular carcinoma tumors in mice [82,83]. The conjugates of humanized G7 monoclonal antibody and monomethyl auristatin E (MMAE) also exhibited antitumor activity in HCC-bearing mice [84].

Targeting CD24 has been clinically accomplished in patients with cancer. Notably, ALB9 (Immunotech), a monoclonal antibody specific to CD24, was administered together with an anti-CD21 antibody in patients with B-cell lymphoproliferative disorders after bone marrow or organ transplantation [85]. The participants tolerated the treatment well; however, some experienced immune-related adverse events (irAEs) such as diarrhea and thrombocytopenia [85]. Moreover, all patients exhibited transient neutropenia. This treatment resulted in complete remission in 16 of 26 patients, as it controlled oligoclonal B-cell proliferation. These findings confirmed that treatment with anti-CD24 and anti-CD21 antibodies ensures long-term safety and efficacy in patients with aggressive post-transplantation B-cell lymphoproliferation [86].

CD24 is abundantly expressed and is considered a putative cancer stem cell (CSC) marker in human cancers [87]. Accumulating preclinical and clinical evidence indicates that CD24 is a promising candidate for anticancer therapy. Recently, the interaction of CD24 and Siglec-10 was recognized to promote immune evasion of cancer cells, and anti-CD24 antibody inhibited tumor growth by substantially increasing the phagocytosis of cancer cells by macrophages [56]. Therefore, CD24 should be considered when targeting the CD24–Siglec-10 axis for cancer immunotherapy. Therefore, agents targeting CD24 must be investigated in preclinical and clinical trials against advanced multiple cancers.

**Table 1:** CD24-targeted cancer therapy in preclinical and clinical models.

Format of Therapy	Name	Tumor	Indications	Effects	Reference
Monoclonal antibody	SWA11	Lung adenocarcinoma, ovarian carcinoma	SCID mouse xenograft model of A549 lung or SKOV3ip ovarian cancer cells	Retardation of the growth of lung and ovarian carcinoma xenografts	[69]
		Pancreatic cancer	SCID mouse xenograft model of BxPC3 pancreatic cancer cells	Prevention of tumor growth	[70]
		Colorectal cancer	Nude mouse xenograft model of HT29 colorectal cancer cells	Reduction in tumor growth rate	[71]
		Multiple myeloma	NOD-Rag1 mouse xenograft model of ARP1 MM cells	Inhibition of multiple myeloma progression	[72]
	ALB9	Bladder cancer	Nude mouse xenograft model of metastatic Lul-1 cells	Reduction in lung metastasis and increase in survival rate	[73]
		Breast cancer	SCID mouse xenograft model of MDA-MB-231 cells	Reduction in lung metastasis and increase in survival rate	[74]
		B-lymphoproliferative disorder (BLPD)	Patients presenting with post-transplant BLPD	Complete remission in 16 of the 26 patients	[85,86]
	Clone SN3	Breast cancer	NSG mouse xenograft model of MCF-7 cells	Reduction in tumor growth	[56]
		Mantle cell lymphoma	Co-culture of Mino cells and macrophages	Increase in phagocytosis of Mino cells by macrophages	
	G7mAb	Lung, liver, and colorectal cancer	Nude mouse xenograft model of A549, Huh-7, and HT-29 cells	Inhibition of tumor growth	[75]
Recombinant bispecific antibody	cG7-MICA	Liver cancer	Nude mouse xenograft model of Huh-7 cells	Reduction in tumor volume and improving survival rate	[76]
CAR-T cell therapy	Anti-CD24-CAR	Pancreatic cancer	SCID mouse xenograft model of human patient’s pancreatic adenocarcinoma (PAC)	Slow tumor growth and prolong survival	[78]
		Ovarian cancer	Patient-derived ovarian cancer cells	Specific killing of patient-derived ovarian cancer cells	[79]
ADC (antibody–drug conjugate)	SWA11-dgA	Burkitt’s lymphoma	SCID mouse xenograft model of BL-38 cells	Improvement of survival	[80]
	SWA11-ZZ-PE38	Colon cancer	Athymic nude mouse xenograft model of colorectal cancer cells	Reduction in tumor volume	[81]
	HN-01 G7mAb-doxorubicin	Liver cancer	Balb/c nude mouse xenograft model of Huh7 or BEL-7402 cells	Inhibition of tumor growth	[82,83]
	hG7mAb-vcMMAE	Liver cancer	Balb/c nude mouse xenograft model of Huh7 cells	Inhibition of tumor growth	[84]

## 3. CD200

CD200, initially known as OX-2, is a type I membrane glycoprotein [88] belonging to the immunoglobulin superfamily (IgSF) of proteins [89]. IgSF proteins contain one or more extracellular Ig-like domains that serve as cell-surface receptors that mediate immune reaction [90]. CD200 consists of two extracellular Ig-like domains (an NH_2_-terminal V-like domain and a smaller C2-like domain), a single transmembrane domain, and a cytoplasmic region comprising 19 amino acids [91]. CD200 is expressed in a wide range of normal cells, including myeloid cells, lymphoid cells, neurons, epithelial cells, endothelial cells, cardiomyocytes, and various neoplastic cells [92,93,94]. It interacts with the CD200 receptor (CD200R), which is mainly expressed in myeloid cells, such as macrophages, neutrophils, and dendritic cells, and is also present in lymphoid cells such as NK cells and T cells [95,96]. CD200 regulates myeloid function by recognizing and engaging CD200R, which is expressed on myeloid cells and transmits inhibitory signals [93,97]. Therefore, the CD200–CD200R axis primarily functions as an immunoregulatory signaling pathway with a potential inhibitory signal. 

### 3.1. CD200 Expression in Tumor Cells and Its Effects on Tumor Progression

Highly expressed CD200 plays pro-tumorigenic roles in various malignant tumors, including hematopoietic and solid cancers [98,99]. Moreover, CD200 has been investigated as a prognostic factor because of its notably increased expression in various cancers, including hematopoietic and solid malignancies [100].

CD200 expression can be observed in various hematopoietic cancers, including acute myeloid leukemia (AML), as well as in certain diseases derived from B lymphocytes, such as chronic lymphocytic leukemia (CLL) and hairy cell leukemia (HCL) [101]. A cohort study of patients with AML reported that CD200 expression was related to worse outcomes, highlighting its role as a prognostic factor for AML [102]. Additionally, patients with AML displayed elevated CD200 expression in leukemic cells, and CD200 overexpression was strongly correlated with elevated Foxp3 regulatory T cells, leading to the generation of an immunosuppressive environment [103]. Wong et al. reported that CD200 was expressed in the cells of patients with CLL. Additionally, blocking CD200 increased the killing of CD200^+^ lymphoma cells and CLL patient cells by CD8^+^ cytotoxic T lymphocytes in vitro [104]. Douds et al. and Alapat et al. observed CD200 positivity in approximately 70% of plasma cell myeloma (PCM) cases, suggesting the possibility of CD200 expression as a diagnostic and prognostic factor for PCM [105,106]. Conticello et al. reported that CD200^+^ cells from patients with MM have an active extracellular signal-regulated kinase (ERK) pathway, which contributes to PCM pathogenesis [107]. CD200 is expressed along with PD-1 and CXCL13 in follicular helper T cells (Tfh) in T cell-derived neoplasm [108]. Pangault et al. also reported enhanced CD200 expression in Tfh and B cells from indolent non-Hodgkin’s B-cell lymphoma, which sustains an immunosuppressive milieu by interacting with CD200R in classical dendritic cells [109].

Numerous research groups have extensively investigated the effects of CD200 on cancer development and aggressiveness in various types of solid cancers. *CD200* mRNA expression is reportedly higher in bone, lung, and liver metastatic tissues from patients with aggressive breast cancer than in adjacent noncancerous breast tissues from those with non-metastatic breast cancer [110]. CD200 is overexpressed in 29.7% of non-small-cell lung cancer (NSCLC) patients and 33.3% of patients with lung large-cell neuroendocrine carcinoma (LCNEC), exhibiting a moderate correlation with PD-L1 expression [111]. Tondell et al. demonstrated that CD200 expression was higher in intratumoral CD4^+^ T cells from patients with NSCLC than that in CD4^+^ T cells from normal lungs, and further elucidated the relationship between elevated CD200 expression in lung cancer tissue and reduced survival [112]. Compared with the substantially low CD200 expression level observed in healthy controls, high CD200 expression was observed in peritumoral stroma from patients with HCC [113]. Several studies have examined the effect of CD200 expression on various types of skin cancer. CD200 expression reportedly plays a prometastatic role in cutaneous squamous cell carcinoma (cSCC), and metastasis was induced through upregulation of the cysteine protease cathepsin K (Ctsk) in CD200-positive cSCC [114,115]. Merkel cell carcinoma (MCC), a rare and aggressive form of skin cancer, exhibits positive CD200 expression in 95.5% of tumors [116]. CD200 is overexpressed in various subgroups of human brain tumors, including glioblastoma, medulloblastoma, ependymoma, and neuroblastoma [117,118]. This finding suggests that CD200 overexpression may be crucial in CNS tumor-induced immunosuppression. Patients with poorly differentiated laryngeal cancer, human pancreatic ductal adenocarcinoma (PDAC), and human clear cell renal cell carcinoma (ccRCC) exhibit elevated CD200 expression, suggesting that it may promote immunosuppression as an immune checkpoint [119,120,121]. 

### 3.2. Interaction between CD200 and Its Receptor, CD200R, in the Tumor Microenvironment

**Figure 3:**
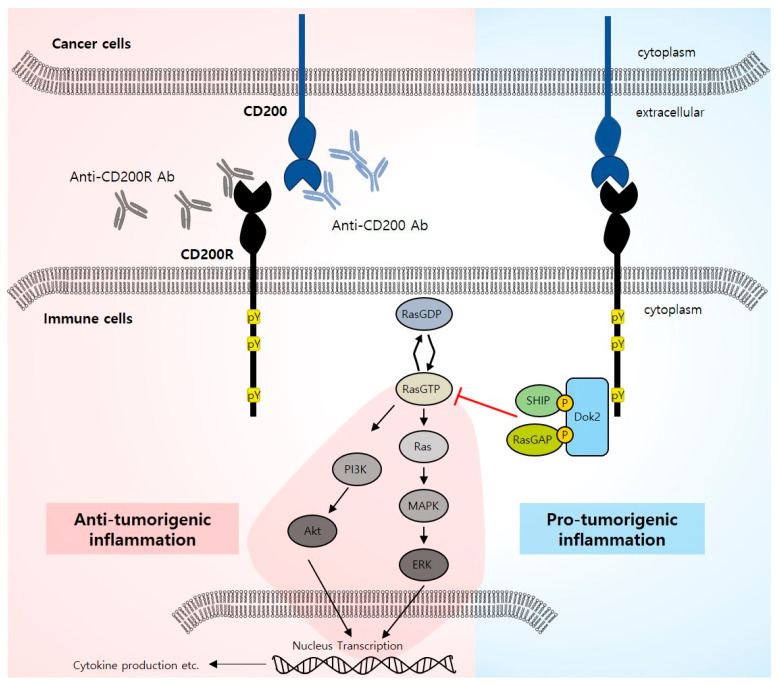
The interaction of CD200 in cancer cells and CD200 receptor in immune cells. CD200 preliminary engages with its CD200 receptor (CD200R), leading to the suppression of immune cell function and activation by inhibiting RAS signaling. CD200–CD200R binding induces phosphorylation of the tyrosine motif in the cytoplasmic tail of CD200R. The adaptor protein tyrosine kinase 2 (DOK-2) is then phosphorylated as a result. Subsequently, this leads to the binding of SH2-containing inositol phosphatase (SHIP) to DOK-2 and the recruitment of Ras GTPase-activating protein (RasGAP). RasGAP inhibits the Ras–MAPK pathway by facilitating GTP hydrolysis from RasGTP to Ras GDP. The RAS signaling pathway further induces the transcription of proinflammatory cytokines. Anti-CD200 antibody or anti-CD200R antibody induces proinflammation by immune cells.

The CD200 receptor (CD200R) has been identified as a novel cognate receptor of CD200 on macrophages, which plays a role in modulating myeloid function [93]. CD200R is a member of the immunoglobulin superfamily (IgSF) of proteins that contains two Ig-like domains [91]. CD200R interacts with the NH2-terminal domain CD200 through its NH2-terminal domain. CD200R possesses a tyrosine motif in its cytoplasmic tail that is phosphorylated when CD200 binds to it. Consequently, the adaptor proteins tyrosine kinase 1 (DOK-1) and tyrosine kinase 2 (DOK-2) are phosphorylated, which consequently results in the binding of SH2-containing inositol phosphatase (SHIP) to DOK-2 and recruits Ras GTPase-activating protein (RasGAP), resulting in the inhibition of the MAPK signaling pathway (Figure 3) [122,123,124]. These processes ultimately lead to the suppression of proinflammatory cytokine release and immune cell activation [122,125]. Downstream signaling of CD200R helps distinguishing it from most other inhibitory receptors that possess an ITIM motif, thereby facilitating inhibition through the engagement of phosphatases [126]. 

CD200R was predominantly observed in cells originating from the myeloid lineage, including macrophages, neutrophils, and dendritic cells. CD200R is also expressed in lymphoid cells, including T, B, and NK cells [93,127]. The interaction between highly expressed CD200 on cancer cells and CD200R on immune cells protects tumor cells by inhibiting myeloid cells [102,128]. *CD200R* mRNA is highly expressed in infiltrating T cells, including T helper (Th) and regulatory T (Treg) cells, in classical Hodgkin lymphoma (cHL) [129]. Additionally, the treatment of anti-CD200R resulted in an increase in IL-2 and TNF-α-positive T cells, providing evidence that the CD200–CD200R axis suppresses T-cell activity in cHL. Vathiotis et al. conducted a study on human lung cancer and observed that among 455 patients with NSCLC, 25% exhibited CD200R overexpression, particularly in the stromal regions of patients with squamous differentiation [111]. This suggests that the CD200–CD200R axis plays a role as an immune checkpoint for patients with NSCLC. Furthermore, CD200R is reportedly overexpressed and co-expressed with multiple immune checkpoints, including PD-1, CTLA-4, and TIM-3, in tumor-infiltrating T cells in NSCLC tumor tissues [130]. These findings suggest that CD200R could function as a biomarker for T-cell phenotypic alterations as well as a potential target for immune therapy.

In patients with HCC, CD200R was predominantly expressed in infiltrating macrophages, along with CD200 expression in the intratumoral region [113]. This combination contributes to a severe malignant condition that leads to lower overall and recurrence-free survival rates compared with that in patients with low CD200R expression. Investigation of the CD200–CD200R interaction in a murine breast cancer model revealed that deficiency of CD200R expression resulted in a reduction in tumor-infiltrating cytotoxic T cells and an increase in the release of inflammatory cytokines, including TNF-α and IL-6 [131]. According to the findings of Owens et al., the interaction between CD200 on SCC keratinocytes and CD200R on myeloid-derived suppressor cells promotes the metastasis of SCC [114,115]. In addition, Liao et al. demonstrated that tumor growth may depend on the relative affinities of the interaction between CD200 and CD200R on M2-type macrophages compared with those on M1-type macrophages [132]. 

CD200 and CD200R are highly expressed in CD83^+^ monocyte-derived dendritic cells (Mo-DCs) stimulated with autologous cancer cell lysates from patients with laryngeal cancer compared with unstimulated Mo-DCs [133]. The proportion of T lymphocytes expressing CD200 is higher in patients with gastric cancer than that in healthy controls. Conversely, the proportion of T lymphocytes expressing CD200R was lower than that of the control group [134]. These findings highlight the potential regulatory influence of the CD200–CD200R axis on the T lymphocyte-dependent immune response in gastric cancer.

CD200–CD200R interaction shares some similarities with the CD47–SIRPα interaction. When CD47 binds to SIRPα, it acts as a signaling mechanism that inhibits myeloid phagocytosis of the “self”, consequently impeding the elimination of cancer cells by phagocytes [135]. CD200, similar to CD47, is a broadly expressed glycoprotein of the immunoglobulin superfamily in various cell types [92]. In contrast, CD200R expression is restricted to immune cells, particularly myeloid cells [127]. CD200 regulates tumor immunity by interacting with the inhibitory receptor CD200R within the tumor microenvironment. Collectively, the CD200–CD200 axis may possess immunotherapeutic potential and is emerging as an innate immune checkpoint in cancer.

### 3.3. CD200–CD200R Pathway as an Immune-Therapeutic Target in Cancers

The CD200–CD200R pathway has gained considerable attention as a crucial target for cancer immunotherapy, primarily because the interaction between CD200 and CD200R assists cancer immune evasion by suppressing immune activity against cancer. The CD200–CD200R axis can be targeted as an immunoregulatory protein in cancer therapy. The interaction between CD200 and CD200R can reportedly be modulated by blocking these interactions using monoclonal antibodies and fusion proteins (Table 2). This targeted approach was aimed at enhancing the immune response in tumor models. 

A growing body of evidence indicates that the therapeutic application of anti-CD200 treatment could potentially provide benefits in CD200-overexpressing malignancies encompassing hematopoietic tumors, such as chronic lymphocytic leukemia (CLL), as well as multiple solid tumors. A study conducted in the early 2000s demonstrated that CD200 is associated with immune rejection of leukemic tumor cells [136]. CD200Fc, which links the extracellular domain of CD200 with the murine IgG2a Fc region, effectively inhibits resistance to tumor growth in CD80-transfected EL4 or C1498 leukemia tumor cell allograft mice. Mice immunized with leukemic tumor cells that overexpress CD80, a costimulatory molecule that plays a role in T-cell activation, exhibit resistance to tumor growth [137]. Additionally, the coinfusion of CD200Fc with CD200R^+^ macrophages further augmented tumor growth suppression. Kretz-Rommel et al. reported that an anti-CD200 antibody inhibits tumor growth in mice bearing CD200-expressing human B-CLL tumors [128]. Oda et al. engineered CD200R immunomodulatory fusion proteins (IFPs) to target CD200 in leukemia [138]. This fusion protein was engineered by replacing the cytoplasmic tail of CD200R with the signaling domain of the costimulatory receptor CD28. Mice with leukemia injected with CD200^+^ FBL (B6 Friend virus-induced erythroleukemia) cells exhibited improved survival when treated with T cells transduced with the CD200R–CD28 fusion protein. The survival rate was higher in mice treated with these modified T cells than that in mice treated with control T cells. 

Samalizumab (Alexion), a recombinant humanized monoclonal antibody that specifically binds to CD200, has been investigated clinically for patients with CLL and MM, and it resulted in a decreased tumor burden in 60% of patients with CLL [139]. Among the 26 study participants, 2 experienced treatment-emergent serious adverse events related to the drug, but these events did not lead to treatment discontinuation [139]. Additionally, certain participants encountered irAEs such as a skin rash and diarrhea; however, these were not clinically significant, indicating a favorable safety profile. These findings support the promotion of antitumor activity through the blockade of the immune checkpoint ligand CD200, resulting in a dose-dependent decrease in CD200 overexpression in CLL cells owing to the binding of samalizumab to CD200.

The synthesized CD200R antagonist peptide (A26059) inhibited the expansion of myeloid-derived suppressor cells (MDSCs), contributing to immune suppression via the CD200–CD200R pathway. The survival of GL261 glioma tumor-bearing mice increased [117], suggesting that disrupting the binding between CD200 and its receptor, CD200R, can potentially enhance the efficacy of an immune-mediated antitumor approach for brain tumors. Another study investigated the effect of CD200 on tumor progression using a metastasis model in rats transplanted with glioma cells. Wistar rats transplanted with C6 glioma cells expressing truncated CD200, which lacks the sequence for CD200R binding, revealed lung metastasis in 44% of the total individuals, whereas rats transplanted with C6 cells expressing full-length CD200 progressed to lung metastasis in all cases [140]. CD200-overexpressing transgenic mice (CD200^tg^ mice) exhibit accelerated tumor growth following injection of EMT6 breast tumor cell lines compared with that in control mice [141]. Blockade of CD200 expression by an anti-CD200 monoclonal antibody attenuated tumor volume in mice injected with EMT6 breast tumors and decreased tumor metastasis [141,142]. Furthermore, the metastasis of EMT6 cells was inhibited in both CD200KO and CD200R1KO mice [143]. Gorczynski et al. reported that administration of an anti-CD200R1 antibody resulted in no EMT6 tumor metastasis in mice that underwent surgical resection of tumors and were subsequently immunized with EMT6 cells [144]. Anti-CD200R1 monoclonal Ab inhibits tumor volume in mice inoculated with murine Hepa1–6 cells, suggesting that the CD200–CD200R pathway is involved in HCC tumor growth [145]. Recently, mice transplanted with head and neck squamous cell carcinoma (HNSCC) cells overexpressing CD200 exhibited attenuated tumor growth when treated with adenovirus-expressing soluble CD200R-Ig [146]. In addition, adenovirus-expressing soluble CD200R-Ig eliminated the pro-tumor effects of CD200, including the induction of M2-like polarization, increased recruitment of regulatory T cells, and decreased the number of CD8^+^ T cells. Accumulating preclinical and clinical studies have shown that targeting the CD200–CD200R axis could effectively enhance antitumor immune responses.

**Table 2:** Cancer therapy targeting CD200–CD200R axis in preclinical and clinical model.

Format of Therapy	Name	Tumors	Indications	Effects	Reference
Antibody	CD200Fc	Leukemic tumor	CD80-transfected EL4 or C1498 leukemia tumor cell allograft mice	Inhibition of tumor growth protection	[136]
	Anti-CD200 Ab	B-cell chronic lymphocytic leukemia	Mice bearing CD200-expressing Namalwa tumor cells	Inhibition of tumor growth	[128]
	Samalizumab (recombinant humanized monoclonal antibody that targets CD200)	Chronic lymphocytic leukemia (CLL) and multiple myeloma (MM)	23 patients with advanced CLL and 3 patients with MM (Phase I study: NCT00648739)	Decrease in tumor burden in 14 CLL patients	[139]
	Rat anti-mouse CD200 Ab	Breast tumor	EMT6 tumor cells injected into CD200^tg^, CD200^KO^, and CD200R1^KO^ mice	Inhibition of tumor growth and metastasis in tumor-bearing mice by anti-CD200 Ab; inhibition of tumor metastasis in CD200KO and CD200R1KO mice	[141,142,143]
	Rabbit Fab anti-CD200R1 Ab	Breast tumor	Mice that underwent surgical resection of the tumors were immunized with EMT6 tumor cells	No EMT6 tumor metastasis in mice immunized with EMT6 tumor cells	[144]
	Anti-CD200R1 mAb	Liver cancer	Murine hepatoma cell line, Hepa1–6 cells, inoculated mice	Inhibition of tumor growth in Hepa1–6-inoculated mice by anti-CD200R1 mAb	[145]
	Adenovirus-expressing sCD200R1-Ig (fusion of the soluble extracellular domain of CD200R1 and Fc domain of mouse IgG2a) (Ad5sCD200R1)	Head and neck cancer	Mice injected with CD200-overexpressing HNSCC cells	Inhibition of tumor growth in mice injected with CD200-overexpressing HNSCC cells by Ad5sCD200R1	[146]
CD200R antagonist peptide	A26059	Glioma tumor	GL261 glioma tumor-implanted mice	Inhibition of tumor growth and increased survival in tumor-bearing mice	[117]
	Truncated CD200 that lacks the part for CD200R binding	Glioma tumor	Truncated CD200 (lack of CD200R binding part) or full-length CD200-expressing C6 glioma cell-transplanted Wistar rats	Inhibition of lung metastasis in rats transplanted with C6 cells expressing truncated CD200	[140]
Immunomodulatory fusion protein	CD200R–CD28 IFP	Murine leukemia	FBL mouse model of disseminated leukemia	Enhancement of survival of murine leukemia mouse model injected with CD200^+^ FBL cells	[138]

## 4. Conclusions and Perspectives

The effective outcomes observed in clinical trials of immune checkpoint inhibitors emphasize the vital role of the immune system in cancer management. Immune checkpoint inhibitors have emerged as the standard treatment for several malignancies because they can restore and strengthen the anticancer immune response. While certain tumor types have exhibited impressive effectiveness with certain immune checkpoint inhibitors, most patients still demonstrate resistance [17]. Currently, the efficacy of immune checkpoint therapies is unsatisfactory. Extensive explorations have been conducted to identify predictors and biomarkers of cancer immunotherapy employing ICIs [147,148]. The efficacy of ICIs has been comprehensively evaluated through the identification of multiple biomarkers, incorporating recent data on the tumor genome and neoantigen biomarkers, the immune microenvironment phenotype of the tumor, host-related factors, and markers obtained through liquid biopsies. Importantly, the investigation of additional molecules involved in immune checkpoint regulation has garnered attention. Notably, the overexpression of immune checkpoint molecules in cancer cells, along with their corresponding receptors on myeloid lineage cells, such as macrophages and dendritic cells, has underscored the significance of innate immune checkpoints in harnessing the antitumor immune response. The CD47–SIRPα signaling axis is a well-known innate immune checkpoint, and blocking this interaction serves as a critical regulator of macrophage phagocytosis and activation [135]. In the present review, we propose that the CD24–Siglec-10 and CD200–CD200R axes are emerging immune checkpoints. Recently, increasing evidence has proven that CD24, highly expressed in multiple cancer cells, serves as a “Don’t eat me” signal and modulates macrophage activity in concert with Siglec-10. Therefore, blocking the interaction between CD24 and Siglec-10 may improve the host immune response against cancer cells by targeting phagocytic checkpoints in cancer immunotherapy. CD200 is an immune checkpoint molecule that suppresses innate immune cell activation by interacting with CD200R. CD200 is highly expressed in various malignant tumor cells and has a pro-tumor effect [98]. The receptor for CD200, CD200R, is predominantly expressed in myeloid cells, including macrophages [127]. The impact of the CD200–CD200R axis on tumor growth and progression has been confirmed in various tumor microenvironments. 

Collectively, several preclinical and clinical studies have demonstrated the significance of the CD24–Siglec-10 and CD200–CD200R axes as targets for immune checkpoint blockade. Although no drugs have entered the clinical stages, the effectiveness of targeting these two immune checkpoint axes needs to be investigated in further clinical studies across multiple cancer types and combinatorial studies with other chemotherapy and diverse immunotherapy approaches in the near future. Blocking other immune checkpoint molecules, such as PD-1 and CTLA-4, along with CD24 or CD200, may synergistically enhance antitumor immunity. Based on the current knowledge of CD24 and CD200, further research suggests that CD24- and CD200-targeted treatments are potential immunotherapeutic drugs for patients with cancer. 

## Data Availability

Not applicable.

## References

[1] Bray F., Laversanne M., Weiderpass E., Soerjomataram I. (2021). The ever-increasing importance of cancer as a leading cause of premature death worldwide. Cancer.

[2] Siegel R.L., Miller K.D., Jemal A. (2020). Cancer statistics, 2020. CA Cancer J. Clin..

[3] Ribas A., Wolchok J.D. (2018). Cancer immunotherapy using checkpoint blockade. Science.

[4] Gun S.Y., Lee S.W.L., Sieow J.L., Wong S.C. (2019). Targeting immune cells for cancer therapy. Redox Biol..

[5] Darvin P., Toor S.M., Sasidharan Nair V., Elkord E. (2018). Immune checkpoint inhibitors: Recent progress and potential biomarkers. Exp. Mol. Med..

[6] Wei S.C., Duffy C.R., Allison J.P. (2018). Fundamental mechanisms of immune checkpoint blockade therapy. Cancer Discov..

[7] Haanen J.B., Robert C. (2015). Immune checkpoint inhibitors. Prog. Tumor Res..

[8] Liu X., Hogg G.D., DeNardo D.G. (2021). Rethinking immune checkpoint blockade: ‘Beyond the t cell’. J. Immunother. Cancer.

[9] Marin-Acevedo J.A., Kimbrough E.O., Lou Y. (2021). Next generation of immune checkpoint inhibitors and beyond. J. Hematol. Oncol..

[10] Lentz R.W., Colton M.D., Mitra S.S., Messersmith W.A. (2021). Innate immune checkpoint inhibitors: The next breakthrough in medical oncology?. Mol. Cancer Ther..

[11] Chen D.S., Mellman I. (2013). Oncology meets immunology: The cancer-immunity cycle. Immunity.

[12] Feng M., Jiang W., Kim B.Y.S., Zhang C.C., Fu Y.X., Weissman I.L. (2019). Phagocytosis checkpoints as new targets for cancer immunotherapy. Nat. Rev. Cancer.

[13] Liu X., Kwon H., Li Z., Fu Y.X. (2017). Is CD47 an innate immune checkpoint for tumor evasion?. J. Hematol. Oncol..

[14] Zhang W., Huang Q., Xiao W., Zhao Y., Pi J., Xu H., Zhao H., Xu J., Evans C.E., Jin H. (2020). Advances in anti-tumor treatments targeting the CD47/sirpalpha axis. Front. Immunol..

[15] Li Z., Li Y., Gao J., Fu Y., Hua P., Jing Y., Cai M., Wang H., Tong T. (2021). The role of CD47-sirpalpha immune checkpoint in tumor immune evasion and innate immunotherapy. Life Sci..

[16] Wang Y., Zhao C., Liu Y., Wang C., Jiang H., Hu Y., Wu J. (2022). Recent advances of tumor therapy based on the CD47-sirpalpha axis. Mol. Pharm..

[17] Bagchi S., Yuan R., Engleman E.G. (2021). Immune checkpoint inhibitors for the treatment of cancer: Clinical impact and mechanisms of response and resistance. Annu. Rev. Pathol..

[18] Velcheti V., Schalper K. (2016). Basic overview of current immunotherapy approaches in cancer. Am. Soc. Clin. Oncol. Educ. Book.

[19] Fang X., Zheng P., Tang J., Liu Y. (2010). Cd24: From a to z. Cell. Mol. Immunol..

[20] Eyvazi S., Kazemi B., Dastmalchi S., Bandehpour M. (2018). Involvement of CD24 in multiple cancer related pathways makes it an interesting new target for cancer therapy. Curr. Cancer Drug Targets.

[21] Hardy R.R., Hayakawa K., Parks D.R., Herzenberg L.A., Herzenberg L.A. (1984). Murine b cell differentiation lineages. J. Exp. Med..

[22] Ayre D.C., Pallegar N.K., Fairbridge N.A., Canuti M., Lang A.S., Christian S.L. (2016). Analysis of the structure, evolution, and expression of CD24, an important regulator of cell fate. Gene.

[23] Li O., Zheng P., Liu Y. (2004). Cd24 expression on t cells is required for optimal t cell proliferation in lymphopenic host. J. Exp. Med..

[24] Nielsen P.J., Lorenz B., Muller A.M., Wenger R.H., Brombacher F., Simon M., von der Weid T., Langhorne W.J., Mossmann H., Kohler G. (1997). Altered erythrocytes and a leaky block in b-cell development in CD24/hsa-deficient mice. Blood.

[25] Baumann P., Cremers N., Kroese F., Orend G., Chiquet-Ehrismann R., Uede T., Yagita H., Sleeman J.P. (2005). Cd24 expression causes the acquisition of multiple cellular properties associated with tumor growth and metastasis. Cancer Res..

[26] Lee J.H., Kim S.H., Lee E.S., Kim Y.S. (2009). Cd24 overexpression in cancer development and progression: A meta-analysis. Oncol. Rep..

[27] Bradley C.A. (2019). Cd24—A novel ‘don’t eat me’ signal. Nat Rev. Cancer.

[28] Ni Y.H., Zhao X., Wang W. (2020). Cd24, a review of its role in tumor diagnosis, progression and therapy. Curr. Gene Ther..

[29] Panagiotou E., Syrigos N.K., Charpidou A., Kotteas E., Vathiotis I.A. (2022). Cd24: A novel target for cancer immunotherapy. J. Pers. Med..

[30] Fogel M., Friederichs J., Zeller Y., Husar M., Smirnov A., Roitman L., Altevogt P., Sthoeger Z.M. (1999). Cd24 is a marker for human breast carcinoma. Cancer Lett..

[31] Athanassiadou P., Grapsa D., Gonidi M., Athanassiadou A.M., Tsipis A., Patsouris E. (2009). Cd24 expression has a prognostic impact in breast carcinoma. Pathol. Res. Pract..

[32] Kristiansen G., Winzer K.J., Mayordomo E., Bellach J., Schluns K., Denkert C., Dahl E., Pilarsky C., Altevogt P., Guski H. (2003). Cd24 expression is a new prognostic marker in breast cancer. Clin. Cancer Res..

[33] Hosonaga M., Arima Y., Sugihara E., Kohno N., Saya H. (2014). Expression of CD24 is associated with her2 expression and supports her2-akt signaling in her2-positive breast cancer cells. Cancer Sci..

[34] Kim H.J., Kim M.J., Ahn S.H., Son B.H., Kim S.B., Ahn J.H., Noh W.C., Gong G. (2011). Different prognostic significance of CD24 and CD44 expression in breast cancer according to hormone receptor status. Breast.

[35] Surowiak P., Materna V., Paluchowski P., Matkowski R., Wojnar A., Maciejczyk A., Pudelko M., Kornafel J., Dietel M., Kristiansen G. (2006). Cd24 expression is specific for tamoxifen-resistant ductal breast cancer cases. Anticancer Res..

[36] Kristiansen G., Denkert C., Schluns K., Dahl E., Pilarsky C., Hauptmann S. (2002). Cd24 is expressed in ovarian cancer and is a new independent prognostic marker of patient survival. Am. J. Pathol..

[37] Nakamura K., Terai Y., Tanabe A., Ono Y.J., Hayashi M., Maeda K., Fujiwara S., Ashihara K., Nakamura M., Tanaka Y. (2017). Cd24 expression is a marker for predicting clinical outcome and regulates the epithelial-mesenchymal transition in ovarian cancer via both the akt and erk pathways. Oncol. Rep..

[38] Ono Y.J., Tanabe A., Tanaka T., Tanaka Y., Hayashi M., Terai Y., Ohmichi M. (2015). Met signaling cascade is amplified by the recruitment of phosphorylated met to lipid rafts via CD24 and leads to drug resistance in endometrial cancer cell lines. Mol. Cancer Ther..

[39] Kwon G.Y., Ha H., Ahn G., Park S.Y., Huh S.J., Park W. (2007). Role of CD24 protein in predicting metastatic potential of uterine cervical squamous cell carcinoma in patients treated with radiotherapy. Int. J. Radiat. Oncol. Biol. Phys..

[40] Sano A., Kato H., Sakurai S., Sakai M., Tanaka N., Inose T., Saito K., Sohda M., Nakajima M., Nakajima T. (2009). Cd24 expression is a novel prognostic factor in esophageal squamous cell carcinoma. Ann. Surg. Oncol..

[41] Fujikuni N., Yamamoto H., Tanabe K., Naito Y., Sakamoto N., Tanaka Y., Yanagihara K., Oue N., Yasui W., Ohdan H. (2014). Hypoxia-mediated CD24 expression is correlated with gastric cancer aggressiveness by promoting cell migration and invasion. Cancer Sci..

[42] Bektas S., Bahadir B., Ucan B.H., Ozdamar S.O. (2010). Cd24 and galectin-1 expressions in gastric adenocarcinoma and clinicopathologic significance. Pathol. Oncol. Res..

[43] Darwish N.S., Kim M.A., Chang M.S., Lee H.S., Lee B.L., Kim Y.I., Kim W.H. (2004). Prognostic significance of CD24 expression in gastric carcinoma. Cancer Res. Treat..

[44] Choi D., Lee H.W., Hur K.Y., Kim J.J., Park G.S., Jang S.H., Song Y.S., Jang K.S., Paik S.S. (2009). Cancer stem cell markers CD133 and CD24 correlate with invasiveness and differentiation in colorectal adenocarcinoma. World J. Gastroenterol..

[45] Majores M., Schindler A., Fuchs A., Stein J., Heukamp L., Altevogt P., Kristiansen G. (2015). Membranous CD24 expression as detected by the monoclonal antibody swa11 is a prognostic marker in non-small cell lung cancer patients. BMC Clin. Pathol..

[46] Yang X.R., Xu Y., Yu B., Zhou J., Li J.C., Qiu S.J., Shi Y.H., Wang X.Y., Dai Z., Shi G.M. (2009). Cd24 is a novel predictor for poor prognosis of hepatocellular carcinoma after surgery. Clin. Cancer Res..

[47] Ikenaga N., Ohuchida K., Mizumoto K., Yu J., Kayashima T., Hayashi A., Nakata K., Tanaka M. (2010). Characterization of CD24 expression in intraductal papillary mucinous neoplasms and ductal carcinoma of the pancreas. Hum. Pathol..

[48] Jacob J., Bellach J., Grutzmann R., Alldinger I., Pilarsky C., Dietel M., Kristiansen G. (2004). Expression of CD24 in adenocarcinomas of the pancreas correlates with higher tumor grades. Pancreatology.

[49] Ahmed M.A., Al-Attar A., Kim J., Watson N.F., Scholefield J.H., Durrant L.G., Ilyas M. (2009). Cd24 shows early upregulation and nuclear expression but is not a prognostic marker in colorectal cancer. J. Clin. Pathol..

[50] Sagiv E., Memeo L., Karin A., Kazanov D., Jacob-Hirsch J., Mansukhani M., Rechavi G., Hibshoosh H., Arber N. (2006). Cd24 is a new oncogene, early at the multistep process of colorectal cancer carcinogenesis. Gastroenterology.

[51] Pieper K., Grimbacher B., Eibel H. (2013). B-cell biology and development. J. Allergy Clin. Immunol..

[52] Lebel E., Nachmias B., Pick M., Gross Even-Zohar N., Gatt M.E. (2022). Understanding the bioactivity and prognostic implication of commonly used surface antigens in multiple myeloma. J. Clin. Med..

[53] Higashi M., Momose S., Takayanagi N., Tanaka Y., Anan T., Yamashita T., Kikuchi J., Tokuhira M., Kizaki M., Tamaru J.I. (2022). Cd24 is a surrogate for ‘immune-cold’ phenotype in aggressive large b-cell lymphoma. J. Pathol. Clin. Res..

[54] Huang P.Y., Best O.G., Almazi J.G., Belov L., Davis Z.A., Majid A., Dyer M.J., Pascovici D., Mulligan S.P., Christopherson R.I. (2014). Cell surface phenotype profiles distinguish stable and progressive chronic lymphocytic leukemia. Leuk. Lymphoma.

[55] Dunn G.P., Old L.J., Schreiber R.D. (2004). The three es of cancer immunoediting. Annu. Rev. Immunol..

[56] Barkal A.A., Brewer R.E., Markovic M., Kowarsky M., Barkal S.A., Zaro B.W., Krishnan V., Hatakeyama J., Dorigo O., Barkal L.J. (2019). Cd24 signalling through macrophage Siglec-10 is a target for cancer immunotherapy. Nature.

[57] Macauley M.S., Crocker P.R., Paulson J.C. (2014). Siglec-mediated regulation of immune cell function in disease. Nat. Rev. Immunol..

[58] Daeron M., Jaeger S., Du Pasquier L., Vivier E. (2008). Immunoreceptor tyrosine-based inhibition motifs: A quest in the past and future. Immunol. Rev..

[59] Neel B.G., Gu H., Pao L. (2003). The ‘shp’ing news: Sh2 domain-containing tyrosine phosphatases in cell signaling. Trends Biochem. Sci..

[60] Pillai S., Netravali I.A., Cariappa A., Mattoo H. (2012). Siglecs and immune regulation. Annu. Rev. Immunol..

[61] Li N., Zhang W., Wan T., Zhang J., Chen T., Yu Y., Wang J., Cao X. (2001). Cloning and characterization of Siglec-10, a novel sialic acid binding member of the ig superfamily, from human dendritic cells. J. Biol. Chem..

[62] Duan S., Paulson J.C. (2020). Siglecs as immune cell checkpoints in disease. Annu. Rev. Immunol..

[63] Chen G.Y., Brown N.K., Zheng P., Liu Y. (2014). Siglec-g/10 in self-nonself discrimination of innate and adaptive immunity. Glycobiology.

[64] Freile J.A., Ustyanovska Avtenyuk N., Corrales M.G., Lourens H.J., Huls G., van Meerten T., Cendrowicz E., Bremer E. (2022). Cd24 is a potential immunotherapeutic target for mantle cell lymphoma. Biomedicines.

[65] Zou K.L., Lan Z., Cui H., Zhao Y.Y., Wang W.M., Yu G.T. (2022). Cd24 blockade promotes anti-tumor immunity in oral squamous cell carcinoma. Oral Dis..

[66] Li W., Wang F., Guo R., Bian Z., Song Y. (2022). Targeting macrophages in hematological malignancies: Recent advances and future directions. J. Hematol. Oncol..

[67] Xiao N., Zhu X., Li K., Chen Y., Liu X., Xu B., Lei M., Xu J., Sun H.C. (2021). Blocking siglec-10^hi^ tumor-associated macrophages improves anti-tumor immunity and enhances immunotherapy for hepatocellular carcinoma. Exp. Hematol. Oncol..

[68] Zahavi D., Weiner L. (2020). Monoclonal antibodies in cancer therapy. Antibodies.

[69] Salnikov A.V., Bretz N.P., Perne C., Hazin J., Keller S., Fogel M., Herr I., Schlange T., Moldenhauer G., Altevogt P. (2013). Antibody targeting of CD24 efficiently retards growth and influences cytokine milieu in experimental carcinomas. Br. J. Cancer.

[70] Bretz N.P., Salnikov A.V., Perne C., Keller S., Wang X., Mierke C.T., Fogel M., Erbe-Hofmann N., Schlange T., Moldenhauer G. (2012). Cd24 controls src/stat3 activity in human tumors. Cell. Mol. Life Sci..

[71] Sagiv E., Starr A., Rozovski U., Khosravi R., Altevogt P., Wang T., Arber N. (2008). Targeting CD24 for treatment of colorectal and pancreatic cancer by monoclonal antibodies or small interfering rna. Cancer Res..

[72] Gao M., Bai H., Jethava Y., Wu Y., Zhu Y., Yang Y., Xia J., Cao H., Franqui-Machin R., Nadiminti K. (2020). Identification and characterization of tumor-initiating cells in multiple myeloma. J. Natl. Cancer Inst..

[73] Overdevest J.B., Thomas S., Kristiansen G., Hansel D.E., Smith S.C., Theodorescu D. (2011). Cd24 offers a therapeutic target for control of bladder cancer metastasis based on a requirement for lung colonization. Cancer Res..

[74] Chan S.H., Tsai K.W., Chiu S.Y., Kuo W.H., Chen H.Y., Jiang S.S., Chang K.J., Hung W.C., Wang L.H. (2019). Identification of the novel role of CD24 as an oncogenesis regulator and therapeutic target for triple-negative breast cancer. Mol. Cancer Ther..

[75] Chen Z., Wang T., Tu X., Xie W., He H., Wang M., Zhang J. (2017). Antibody-based targeting of CD24 enhances antitumor effect of cetuximab via attenuating phosphorylation of src/stat3. Biomed. Pharmacother..

[76] Han Y., Sun F., Zhang X., Wang T., Jiang J., Cai J., Gao Q., Hezam K., Liu Y., Xie J. (2019). Cd24 targeting bi-specific antibody that simultaneously stimulates nkg2d enhances the efficacy of cancer immunotherapy. J. Cancer Res. Clin. Oncol..

[77] Newick K., O’Brien S., Moon E., Albelda S.M. (2017). Car t cell therapy for solid tumors. Annu. Rev. Med..

[78] Maliar A., Servais C., Waks T., Chmielewski M., Lavy R., Altevogt P., Abken H., Eshhar Z. (2012). Redirected t cells that target pancreatic adenocarcinoma antigens eliminate tumors and metastases in mice. Gastroenterology.

[79] Klapdor R., Wang S., Morgan M., Dork T., Hacker U., Hillemanns P., Buning H., Schambach A. (2019). Characterization of a novel third-generation anti-CD24-car against ovarian cancer. Int. J. Mol. Sci..

[80] Schnell R., Katouzi A.A., Linnartz C., Schoen G., Drillich S., Hansmann M.L., Schiefer D., Barth S., Zangemeister-Wittke U., Stahel R.A. (1996). Potent anti-tumor effects of an anti-CD24 ricin a-chain immunotoxin in vitro and in a disseminated human burkitt’s lymphoma model in scid mice. Int. J. Cancer.

[81] Shapira S., Shapira A., Starr A., Kazanov D., Kraus S., Benhar I., Arber N. (2011). An immunoconjugate of anti-CD24 and pseudomonas exotoxin selectively kills human colorectal tumors in mice. Gastroenterology.

[82] Sun F., Wang Y., Luo X., Ma Z., Xu Y., Zhang X., Lv T., Zhang Y., Wang M., Huang Z. (2019). Anti-CD24 antibody-nitric oxide conjugate selectively and potently suppresses hepatic carcinoma. Cancer Res..

[83] Ma Z., He H., Sun F., Xu Y., Huang X., Ma Y., Zhao H., Wang Y., Wang M., Zhang J. (2017). Selective targeted delivery of doxorubicin via conjugating to anti-CD24 antibody results in enhanced antitumor potency for hepatocellular carcinoma both in vitro and in vivo. J. Cancer Res. Clin. Oncol..

[84] Sun F., Wang T., Jiang J., Wang Y., Ma Z., Li Z., Han Y., Pan M., Cai J., Wang M. (2017). Engineering a high-affinity humanized anti-CD24 antibody to target hepatocellular carcinoma by a novel CDR grafting design. Oncotarget.

[85] Fischer A., Blanche S., Le Bidois J., Bordigoni P., Garnier J.L., Niaudet P., Morinet F., Le Deist F., Fischer A.M., Griscelli C. (1991). Anti-b-cell monoclonal antibodies in the treatment of severe b-cell lymphoproliferative syndrome following bone marrow and organ transplantation. N. Engl. J. Med..

[86] Benkerrou M., Jais J.P., Leblond V., Durandy A., Sutton L., Bordigoni P., Garnier J.L., Le Bidois J., Le Deist F., Blanche S. (1998). Anti-b-cell monoclonal antibody treatment of severe posttransplant b-lymphoproliferative disorder: Prognostic factors and long-term outcome. Blood.

[87] Zhang S., Zhu N., Li H.F., Gu J., Zhang C.J., Liao D.F., Qin L. (2022). The lipid rafts in cancer stem cell: A target to eradicate cancer. Stem Cell Res. Ther..

[88] Barclay A.N., Ward H.A. (1982). Purification and chemical characterisation of membrane glycoproteins from rat thymocytes and brain, recognised by monoclonal antibody mrc ox 2. Eur. J. Biochem..

[89] McCaughan G.W., Clark M.J., Hurst J., Grosveld F., Barclay A.N. (1987). The gene for mrc ox-2 membrane glycoprotein is localized on human chromosome 3. Immunogenetics.

[90] Buck C.A. (1992). Immunoglobulin superfamily: Structure, function and relationship to other receptor molecules. Semin. Cell Biol..

[91] Hatherley D., Lea S.M., Johnson S., Barclay A.N. (2013). Structures of CD200/CD200 receptor family and implications for topology, regulation, and evolution. Structure.

[92] Barclay A.N., Wright G.J., Brooke G., Brown M.H. (2002). CD200 and membrane protein interactions in the control of myeloid cells. Trends Immunol..

[93] Wright G.J., Puklavec M.J., Willis A.C., Hoek R.M., Sedgwick J.D., Brown M.H., Barclay A.N. (2000). Lymphoid/neuronal cell surface ox2 glycoprotein recognizes a novel receptor on macrophages implicated in the control of their function. Immunity.

[94] Barclay A.N. (1981). Different reticular elements in rat lymphoid tissue identified by localization of ia, thy-1 and mrc ox 2 antigens. Immunology.

[95] Rijkers E.S., de Ruiter T., Baridi A., Veninga H., Hoek R.M., Meyaard L. (2008). The inhibitory CD200R is differentially expressed on human and mouse t and b lymphocytes. Mol. Immunol..

[96] Jenmalm M.C., Cherwinski H., Bowman E.P., Phillips J.H., Sedgwick J.D. (2006). Regulation of myeloid cell function through the CD200 receptor. J. Immunol..

[97] Hoek R.M., Ruuls S.R., Murphy C.A., Wright G.J., Goddard R., Zurawski S.M., Blom B., Homola M.E., Streit W.J., Brown M.H. (2000). Down-regulation of the macrophage lineage through interaction with ox2 (CD200). Science.

[98] Shao A., Owens D.M. (2023). The immunoregulatory protein CD200 as a potentially lucrative yet elusive target for cancer therapy. Oncotarget.

[99] Sorigue M., Magnano L., Miljkovic M.D., Nieto-Moragas J., Santos-Gomez M., Villamor N., Junca J., Morales-Indiano C. (2020). Positive predictive value of CD200 positivity in the differential diagnosis of chronic lymphocytic leukemia. Cytom. B Clin. Cytom..

[100] Moreaux J., Veyrune J.L., Reme T., De Vos J., Klein B. (2008). CD200: A putative therapeutic target in cancer. Biochem. Biophys. Res. Commun..

[101] Staub R.B., Marcondes N.A., Rotta L.N. (2021). CD200 expression in hematopoietic neoplasms: Beyond a marker for diagnosis of b-cell neoplasms. Crit. Rev. Oncol. Hematol..

[102] Tonks A., Hills R., White P., Rosie B., Mills K.I., Burnett A.K., Darley R.L. (2007). CD200 as a prognostic factor in acute myeloid leukaemia. Leukemia.

[103] Memarian A., Nourizadeh M., Masoumi F., Tabrizi M., Emami A.H., Alimoghaddam K., Hadjati J., Mirahmadian M., Jeddi-Tehrani M. (2013). Upregulation of CD200 is associated with foxp3^+^ regulatory t cell expansion and disease progression in acute myeloid leukemia. Tumour Biol..

[104] Wong K.K., Khatri I., Shaha S., Spaner D.E., Gorczynski R.M. (2010). The role of CD200 in immunity to b cell lymphoma. J. Leukoc. Biol..

[105] Douds J.J., Long D.J., Kim A.S., Li S. (2014). Diagnostic and prognostic significance of CD200 expression and its stability in plasma cell myeloma. J. Clin. Pathol..

[106] Alapat D., Coviello-Malle J., Owens R., Qu P., Barlogie B., Shaughnessy J.D., Lorsbach R.B. (2012). Diagnostic usefulness and prognostic impact of CD200 expression in lymphoid malignancies and plasma cell myeloma. Am. J. Clin. Pathol..

[107] Conticello C., Giuffrida R., Parrinello N., Buccheri S., Adamo L., Sciuto M.R., Colarossi C., Aiello E., Chiarenza A., Romano A. (2013). CD200 expression in patients with multiple myeloma: Another piece of the puzzle. Leuk. Res..

[108] Dorfman D.M., Shahsafaei A. (2011). CD200 (ox-2 membrane glycoprotein) is expressed by follicular t helper cells and in angioimmunoblastic t-cell lymphoma. Am. J. Surg. Pathol..

[109] Pangault C., Ame-Thomas P., Rossille D., Dulong J., Caron G., Nonn C., Chatonnet F., Desmots F., Launay V., Lamy T. (2020). Integrative analysis of cell crosstalk within follicular lymphoma cell niche: Towards a definition of the fl supportive synapse. Cancers.

[110] Zhang Y., He W., Zhang S. (2019). Seeking for correlative genes and signaling pathways with bone metastasis from breast cancer by integrated analysis. Front. Oncol..

[111] Vathiotis I.A., MacNeil T., Zugazagoitia J., Syrigos K.N., Aung T.N., Gruver A.M., Vaillancourt P., Hughes I., Hinton S., Driscoll K. (2021). Quantitative assessment of CD200 and CD200R expression in lung cancer. Cancers.

[112] Tondell A., Subbannayya Y., Wahl S.G.F., Flatberg A., Sorhaug S., Borset M., Haug M. (2021). Analysis of intra-tumoral macrophages and t cells in non-small cell lung cancer (nsclc) indicates a role for immune checkpoint and CD200-CD200R interactions. Cancers.

[113] Sun H., Xu J., Huang M., Huang Q., Sun R., Xiao W., Sun C. (2016). CD200R, a co-inhibitory receptor on immune cells, predicts the prognosis of human hepatocellular carcinoma. Immunol. Lett..

[114] Khan I.Z., Del Guzzo C.A., Shao A., Cho J., Du R., Cohen A.O., Owens D.M. (2021). The CD200-CD200R axis promotes squamous cell carcinoma metastasis via regulation of cathepsin k. Cancer Res..

[115] Stumpfova M., Ratner D., Desciak E.B., Eliezri Y.D., Owens D.M. (2010). The immunosuppressive surface ligand CD200 augments the metastatic capacity of squamous cell carcinoma. Cancer Res..

[116] Gaiser M.R., Weis C.A., Gaiser T., Jiang H., Buder-Bakhaya K., Herpel E., Warth A., Xiao Y., Miao L., Brownell I. (2018). Merkel cell carcinoma expresses the immunoregulatory ligand CD200 and induces immunosuppressive macrophages and regulatory t cells. Oncoimmunology.

[117] Moertel C.L., Xia J., LaRue R., Waldron N.N., Andersen B.M., Prins R.M., Okada H., Donson A.M., Foreman N.K., Hunt M.A. (2014). CD200 in cns tumor-induced immunosuppression: The role for CD200 pathway blockade in targeted immunotherapy. J. Immunother. Cancer.

[118] Xin C., Zhu J., Gu S., Yin M., Ma J., Pan C., Tang J., Zhang P., Liu Y., Bai X.F. (2020). CD200 is overexpressed in neuroblastoma and regulates tumor immune microenvironment. Cancer Immunol. Immunother..

[119] Klatka J., Grywalska E., Klatka M., Wasiak M., Andrzejczak A., Rolinski J. (2013). Expression of selected regulatory molecules on the CD83+ monocyte-derived dendritic cells generated from patients with laryngeal cancer and their clinical significance. Eur. Arch. Otorhinolaryngol..

[120] Choueiry F., Torok M., Shakya R., Agrawal K., Deems A., Benner B., Hinton A., Shaffer J., Blaser B.W., Noonan A.M. (2020). CD200 promotes immunosuppression in the pancreatic tumor microenvironment. J. Immunother. Cancer.

[121] Tronik-Le Roux D., Sautreuil M., Bentriou M., Verine J., Palma M.B., Daouya M., Bouhidel F., Lemler S., LeMaoult J., Desgrandchamps F. (2020). Comprehensive landscape of immune-checkpoints uncovered in clear cell renal cell carcinoma reveals new and emerging therapeutic targets. Cancer Immunol. Immunother..

[122] Zhang S., Cherwinski H., Sedgwick J.D., Phillips J.H. (2004). Molecular mechanisms of CD200 inhibition of mast cell activation. J. Immunol..

[123] Mihrshahi R., Brown M.H. (2010). Downstream of tyrosine kinase 1 and 2 play opposing roles in CD200 receptor signaling. J. Immunol..

[124] Lin C.H., Talebian F., Li Y., Zhu J., Liu J.Q., Zhao B., Basu S., Pan X., Chen X., Yan P. (2023). CD200R signaling contributes to unfavorable tumor microenvironment through regulating production of chemokines by tumor-associated myeloid cells. iScience.

[125] Fraser S.D., Sadofsky L.R., Kaye P.M., Hart S.P. (2016). Reduced expression of monocyte CD200R is associated with enhanced proinflammatory cytokine production in sarcoidosis. Sci. Rep..

[126] Mihrshahi R., Barclay A.N., Brown M.H. (2009). Essential roles for dok2 and rasgap in CD200 receptor-mediated regulation of human myeloid cells. J. Immunol..

[127] Wright G.J., Cherwinski H., Foster-Cuevas M., Brooke G., Puklavec M.J., Bigler M., Song Y., Jenmalm M., Gorman D., McClanahan T. (2003). Characterization of the CD200 receptor family in mice and humans and their interactions with CD200. J. Immunol..

[128] Kretz-Rommel A., Qin F., Dakappagari N., Ravey E.P., McWhirter J., Oltean D., Frederickson S., Maruyama T., Wild M.A., Nolan M.J. (2007). CD200 expression on tumor cells suppresses antitumor immunity: New approaches to cancer immunotherapy. J. Immunol..

[129] Wein F., Weniger M.A., Hoing B., Arnolds J., Huttmann A., Hansmann M.L., Hartmann S., Kuppers R. (2017). Complex immune evasion strategies in classical hodgkin lymphoma. Cancer Immunol. Res..

[130] Su Y., Yamazaki S., Morisue R., Suzuki J., Yoshikawa T., Nakatsura T., Tsuboi M., Ochiai A., Ishii G. (2021). Tumor-infiltrating t cells concurrently overexpress CD200R with immune checkpoints pd-1, ctla-4, and tim-3 in non-small-cell lung cancer. Pathobiology.

[131] Erin N., Podnos A., Tanriover G., Duymus O., Cote E., Khatri I., Gorczynski R.M. (2015). Bidirectional effect of CD200 on breast cancer development and metastasis, with ultimate outcome determined by tumor aggressiveness and a cancer-induced inflammatory response. Oncogene.

[132] Liao K.L., Bai X.F., Friedman A. (2013). The role of CD200-CD200R in tumor immune evasion. J. Theor. Biol..

[133] Klatka J., Grywalska E., Klatka M., Rahnama M., Polak A., Rolinski J. (2013). Expression of CD200 and CD200R regulatory molecules on the CD83+ monocyte-derived dendritic cells generated from patients with laryngeal cancer. Folia Histochem. Cytobiol..

[134] Zgodzinski W., Grywalska E., Surdacka A., Zinkiewicz K., Majewski M., Szczepanek D., Wallner G., Rolinski J. (2018). Surface CD200 and CD200R antigens on lymphocytes in advanced gastric cancer: A new potential target for immunotherapy. Arch. Med. Sci..

[135] Matlung H.L., Szilagyi K., Barclay N.A., van den Berg T.K. (2017). The CD47-sirpalpha signaling axis as an innate immune checkpoint in cancer. Immunol. Rev..

[136] Gorczynski R.M., Chen Z., Hu J., Kai Y., Lei J. (2001). Evidence of a role for CD200 in regulation of immune rejection of leukaemic tumour cells in c57bl/6 mice. Clin. Exp. Immunol..

[137] Blazar B.R., Taylor P.A., Boyer M.W., Panoskaltsis-Mortari A., Allison J.P., Vallera D.A. (1997). CD28/B7 interactions are required for sustaining the graft-versus-leukemia effect of delayed post-bone marrow transplantation splenocyte infusion in murine recipients of myeloid or lymphoid leukemia cells. J. Immunol..

[138] Oda S.K., Daman A.W., Garcia N.M., Wagener F., Schmitt T.M., Tan X., Chapuis A.G., Greenberg P.D. (2017). A CD200R-CD28 fusion protein appropriates an inhibitory signal to enhance t-cell function and therapy of murine leukemia. Blood.

[139] Mahadevan D., Lanasa M.C., Farber C., Pandey M., Whelden M., Faas S.J., Ulery T., Kukreja A., Li L., Bedrosian C.L. (2019). Phase i study of samalizumab in chronic lymphocytic leukemia and multiple myeloma: Blockade of the immune checkpoint CD200. J. Immunother. Cancer.

[140] Kuwabara J., Umakoshi A., Abe N., Sumida Y., Ohsumi S., Usa E., Taguchi K., Choudhury M.E., Yano H., Matsumoto S. (2018). Truncated CD200 stimulates tumor immunity leading to fewer lung metastases in a novel wistar rat metastasis model. Biochem. Biophys. Res. Commun..

[141] Gorczynski R.M., Chen Z., Diao J., Khatri I., Wong K., Yu K., Behnke J. (2010). Breast cancer cell CD200 expression regulates immune response to emt6 tumor cells in mice. Breast Cancer Res. Treat..

[142] Gorczynski R.M., Clark D.A., Erin N., Khatri I. (2011). Role of CD200 expression in regulation of metastasis of emt6 tumor cells in mice. Breast Cancer Res. Treat..

[143] Gorczynski R.M., Chen Z., Khatri I., Podnos A., Yu K. (2013). Cure of metastatic growth of emt6 tumor cells in mice following manipulation of CD200:CD200R signaling. Breast Cancer Res. Treat..

[144] Gorczynski R.M., Chen Z., Erin N., Khatri I., Podnos A. (2014). Comparison of immunity in mice cured of primary/metastatic growth of emt6 or 4thm breast cancer by chemotherapy or immunotherapy. PLoS ONE.

[145] Huang S., Pan Y., Zhang Q., Sun W. (2019). Role of CD200/CD200R signaling pathway in regulation of CD4+t cell subsets during thermal ablation of hepatocellular carcinoma. Med. Sci. Monit..

[146] Shin S.P., Goh A.R., Ju J.M., Kang H.G., Kim S.J., Kim J.K., Park E.J., Bae Y.S., Choi K., Jung Y.S. (2021). Local adenoviral delivery of soluble CD200R-ig enhances antitumor immunity by inhibiting CD200-beta-catenin-driven m2 macrophage. Mol. Ther. Oncolytics.

[147] Zelin E., Maronese C.A., Dri A., Toffoli L., Di Meo N., Nazzaro G., Zalaudek I. (2022). Identifying candidates for immunotherapy among patients with non-melanoma skin cancer: A review of the potential predictors of response. J. Clin. Med..

[148] Bai R., Lv Z., Xu D., Cui J. (2020). Predictive biomarkers for cancer immunotherapy with immune checkpoint inhibitors. Biomark. Res..

